# Comprehensive review of One Health systems for emerging infectious disease detection and management

**DOI:** 10.1016/j.onehlt.2025.101253

**Published:** 2025-11-10

**Authors:** Aseel Basheer, Matthew Tran, Baseer Khan, Wolfgang Jentner, Aaron Wendelboe, Jason Vogel, Katrin Kuhn, Michael C. Wimberly, David Ebert

**Affiliations:** a School of Computer Science, University of Oklahoma, OK, US; b Data Institute for Societal Challenges (DISC), The University of Oklahoma, OK, US; c University of Arizona, AZ, US; d College of Public Health, University of Arkansas for Medical Sciences, AR, US; e School of Civil Engineering and Environmental Science, University of Oklahoma, OK, US; f Hudson College of Public Health, University of Oklahoma Health Campus, OK, US; g Department of Geography and Environmental Sustainability, University of Oklahoma, OK, US

**Keywords:** One Health, Emerging infectious disease, Visualization, Dashboards

## Abstract

This systematic review investigates how One Health systems, integrated digital platforms combining human, animal, and environmental health data, are currently designed and implemented for infectious disease detection and management. The study aims to identify integration patterns, functional purposes, and user interactivity across 202 reviewed systems published between 2015 and 2024. It categorized these systems by their purpose, diseases addressed, data types (human, animal, environmental), and user groups, such as public health officials and researchers. The tasks performed include data collection, analysis, visualization, and decision-making. Interactive techniques range from interactive filtering to predictive modeling, with varying levels of user interactivity, and note whether functional systems are available online. The search strategy utilized the keywords “one health dashboard visualization system OR one health dashboard OR one health system” across various databases Including IEEE xplore ScienceDirect PubMed And google scholar As well as other sources. While a significant portion of studies still rely on single-domain data, i.e., 20% of studies use only human data, 12% use only animal data, and 10% use only environmental data. The largest group (30%) integrates human and animal data, followed by 12% combining human and environmental data, and a smaller portion (1%) integrating animal and environmental data. The details of this comprehensive survey can be found on this webpage: https://onlylinks.cc/DjHH. There is a clear trend toward integrating multiple datasets, especially Human and Animal data. However, fully integrated One Health systems that combine all three domains remain relatively limited and often take the form of commentaries rather than applied systems, highlighting an opportunity for more comprehensive, data-driven implementations in future research.

## Highlights

• Reviewed 202 One Health-related papers from different databases.
• Categorized systems mainly by data type, purpose, disease, and user group.
• Found a growing trend in integrating human and animal health data.
• Fully integrated One Health systems remain rare and often non-functional.

## 1 Introduction

The challenges posed by global pandemic, such as COVID-19, and emerging infectious diseases, including H5N1 and mpox, underscore significant public health concerns and the ongoing need for enhanced outbreak management and disease detection [1]. Innovative solutions are essential to address the growing challenges surrounding disease surveillance and pandemic response. Traditional surveillance systems often operate within disciplinary silos, limiting early warning and response capabilities. The One Health approach provides a unifying framework that recognizes the interconnected health of humans, animals, and the environment [2]. It has proven to be a promising approach to addressing public health issues at local, regional, and national levels [3]. Key organizations, including the World Health Organization (WHO) [2] and the Food and Agriculture Organization (FAO) [4], emphasize the One Health approach as a transformative method for managing zoonotic diseases, antimicrobial resistance, vector-borne diseases, and various health challenges. For example, the WHO’s One Health framework emphasizes the importance of integrating human, animal, and environmental health to enhance proactive disease surveillance and response, particularly in the aftermath of the COVID-19 pandemic [5]. The interconnectedness of various health domains highlights the importance of systems that can assist public health officials, researchers, and policymakers in tasks such as early detection, prediction, planning, mitigation, and monitoring [6].

In this study, a *One Health system* refers to a computer-based tool or platform, such as a dashboard, decision-support system, or visualization environment, that integrates human, animal, and environmental health data to assist public health officials, researchers, and policymakers in disease surveillance, prediction, planning, and response [6], [7]. These systems operate within broader institutional and surveillance frameworks; however, they are analyzed here primarily for their technological and computational components.

Over the last decade, the adoption of the One Health approach has spurred the development of visual and analytical systems across regions and disciplines [8]. These systems vary widely in purpose, scope, and interactivity, leveraging diverse datasets to address challenges in outbreak detection, monitoring, and mitigation. However, despite the growing number of such systems, their implementation remains fragmented, and there is limited synthesis of how these systems operationalize the One Health framework in practice.

There are numerous survey papers regarding the One Health approach and its applications, such as [8], [9], [10], [11]. Sharan et al. [9] focused specifically on surveillance strategies for zoonotic diseases, assessing global surveillance frameworks, their design, and the core components necessary for establishing surveillance systems at the human–animal–environment interface. In contrast, Ruegg et al. [8] assessed the effectiveness and integration of One Health initiatives. They employed a structured methodology to assess the impact and coherence of One Health initiatives, with a focus on the evaluation framework rather than the functional features of specific systems.

Bordier et al. [10] addressed surveillance systems in the One Health context, aiming to characterize their organizational and operational aspects rather than their visual or interactive functionalities. Their work seeks to establish a conceptual framework for understanding the essential organizational and functional characteristics of One Health surveillance systems. They explored the factors that impact their implementation, such as inter-sectoral collaboration, and the specific barriers and levers that influence their performance.

Another review by Mukherjee et al. examines the potential of the “One Health” framework as a solution for cross-sectoral challenges, particularly in pathogen detection and managing zoonotic viral and bacterial diseases [11]. This review emphasizes the importance of the “One Health” approach, which connects the detection and effective treatment of diseases by highlighting the interdependent relationship between human and animal health. The paper underscores the vital role that “One Health” initiatives play in preventing and controlling infectious diseases.

Although numerous studies explore the concept of One Health and its significant roles, they tend to focus on specific visual methods and disease characteristics. Although each contributes valuable insights, there remains no comprehensive, systematic review of One Health systems that compares their functional, visual, and analytical characteristics across diseases and domains.

Despite the widespread advocacy for One Health integration, it remains unclear how existing systems implement this concept computationally, specifically, how they combine datasets across domains, support interactivity, and facilitate evidence-based decision-making. The absence of a unified overview of such systems hinders researchers and public health professionals from understanding current capabilities, identifying integration patterns, and recognizing design or data gaps.

To address this gap, this paper presents a comprehensive review of One Health systems and dashboards developed between 2015 and 2024. We systematically categorize 202 studies by purpose, disease focus, data domains (human, animal, environmental), user groups, and visual analytics techniques. By analyzing these dimensions, we aim to identify prevailing trends, assess the maturity of integration across domains, and highlight opportunities for advancing fully integrated, data-driven One Health systems in the management of emerging infectious diseases.

## 2 Materials and methods

**Fig. 1:**
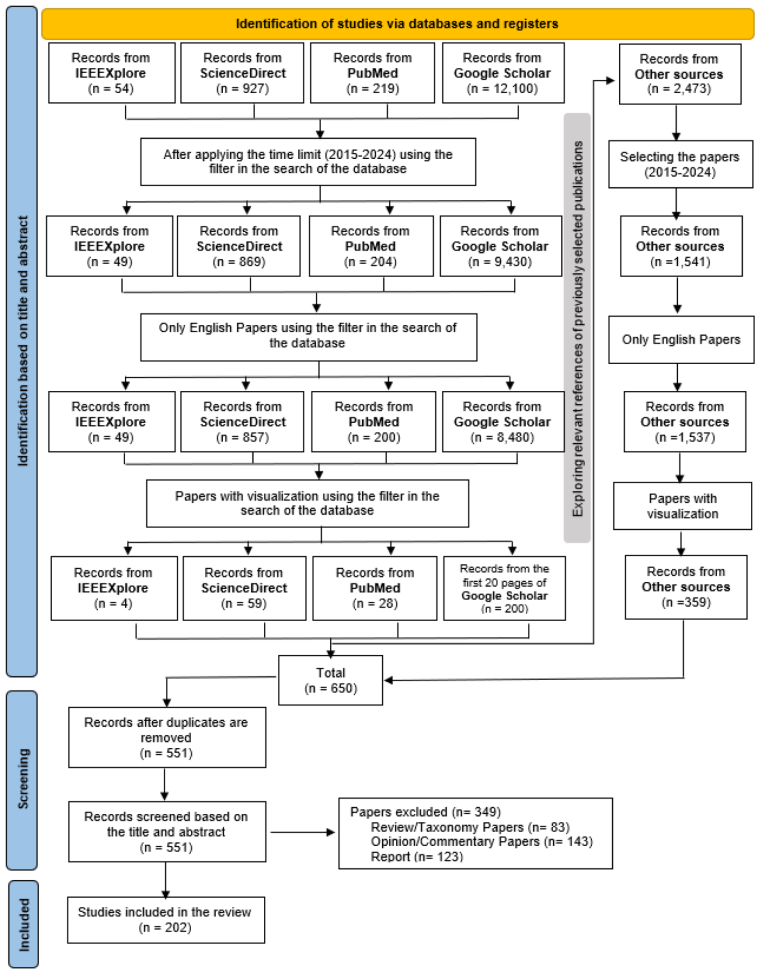
The PRISMA flow diagram.

We followed the Preferred Reporting Items for Systematic Reviews and Meta-Analyses (PRISMA) guidelines [12] to conduct this literature review. The primary research question guiding this study was: “How are One Health systems and dashboards designed and implemented to support infectious disease detection, prediction, planning, mitigation, and monitoring, and what types of data, analytical methods, and user groups do they involve?” As shown in Fig. 1, the systematic review process started with an initial search on IEEEXplore, ScienceDirect, PubMed, and Google Scholar using relevant keywords. After selecting relevant papers from these databases, we followed the “backward citation chasing” approach by looking at the reference lists of selected papers to find earlier relevant studies (cited articles) [13]. Therefore, we examined the references cited in those papers (“other sources”) to identify additional studies.

Four reviewers independently screened all retrieved articles for inclusion based on predefined criteria. Disagreements regarding inclusion or exclusion were resolved through discussion, and when necessary, by consensus among all authors. This collaborative approach helped reduce potential bias in study selection and ensured consistent application of the inclusion criteria. The review process and coding of eligible studies were cross-validated by the team to maintain reliability.

Next, we applied specific criteria to decide which papers could be included in the review. After that, we categorized the selected papers based on various factors, including their purpose, the data they included, and the diseases they addressed. Finally, we concluded the process by analyzing the search statistics to synthesize the results.

### 2.1 Search strategy

A keyword search was conducted using IEEEXplore, ScienceDirect, PubMed, and Google Scholar with the primary search strategy: ”One Health Dashboard Visualization System”, ”One Health Dashboard”, or ”One Health System”. To refine the results, only papers published between 2015 and 2024 (inclusive) were included. Additionally, relevant citations from review papers identified through this search were also considered, provided they pertained to One Health systems. Papers were also gathered from the Surveillance Outbreak Responses Management and Analysis (SORMAS) website [14], specifically those related to the SORMAS system. The focus was primarily on research articles, with review papers being collected to a limited extent.

### 2.2 Inclusion and exclusion criteria

The inclusion criteria for the study were as follows: Systems were required to exhibit a *One Health Focus*, integrating data from at least one of these categories: human health, animal health, and environmental health. An *Infectious Disease Focus* was necessary, targeting the detection, prediction, planning, mitigation, or monitoring of infectious diseases, including the genetic evolution of pathogens, mutations, or species jumping. Systems had to be *Data-Driven*, utilizing datasets such as patient records, veterinary data, or environmental and climate data. In addition, they incorporated *Visualization*, employing techniques like graphs, charts, geospatial maps, network graphs, time-series plots, and hierarchical charts to convey information. Lastly, the systems offered various levels of *User Interaction*, ranging from high to low interactivity. They were required to be based on research publications from the last 10 years (2015–2024) to ensure current relevance.

The exclusion criteria for this study were applied in multiple stages as illustrated in Fig. 1. First, we removed papers published outside the targeted timeline (2015–2024). Next, we excluded papers not published in English. Following this, we excluded papers without a visualization component. Lastly, theoretical papers that described frameworks or proposals without demonstrating a functional system or prototype were not included, nor were bibliographic review papers that overviewed previous publications.

To ensure consistency and reduce selection bias, four independent reviewers screened all articles using these criteria. Any discrepancies were resolved through consensus, and uncertain cases were reviewed collectively by all authors. This collaborative and iterative screening process ensured transparency and adherence to the PRISMA methodology [12].

### 2.3 Classification and data extraction

**Fig. 2:**
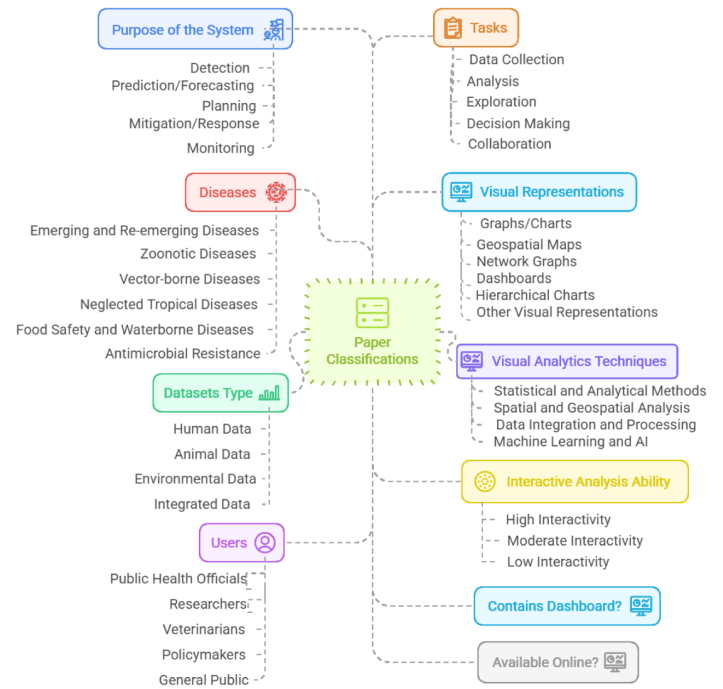
We classified the collected papers in terms of the purpose of the system, diseases, dataset type, users, tasks, visual representations, visual analytics techniques, interactive analysis ability, system or dashboard, and availability.

This systematic review comprehensively analyzed existing systems and dashboards that utilize One Health datasets for infectious disease management, as shown in Fig. 2. The review aimed to identify, categorize, and evaluate key characteristics of these systems, focusing on their purpose, data types, user groups, interactive functionalities, visual representations, analytical techniques, and implementation status.

Each category in the review framework was defined to capture key aspects of disease surveillance and response systems. The *Purpose of the System* addresses what the system or study is designed to achieve. This includes *Detection Systems* that focus on the early detection and surveillance of diseases; *Prediction/Forecasting Systems*, which aim to anticipate disease trends, outbreaks, or pandemics; *Planning Systems*, which support decision-making for future preparedness; *Mitigation/Response Systems*, which are focused on strategies to reduce the impact or respond to outbreaks; and *Monitoring Systems*, which continuously track disease patterns and public health indicators over time.

Another essential categorization involves the type of *diseases* or range of diseases targeted by the system. This includes a range of infectious and non-infectious diseases, such as emerging and re-emerging diseases, zoonotic diseases, and vector-borne diseases. Identifying the disease focus helps assess the applicability and specificity of a system in addressing different public health challenges.

The *datasets* utilized by these systems were classified into several types. *Human Data*, includes patient records, health information, and human mobility data; *Animal Data*, encompasses veterinary data, wildlife tracking, and information on zoonotic diseases; *Environmental Data*, consists of climate data, geospatial data, and other environmental factors; and *Integrated Data*, which combines multiple datasets, including Human, Animal, and Environmental data.

The intended *users* of the system include a broad range of stakeholders, such as public health officials, healthcare providers, researchers, veterinarians, policymakers, and the general public. Understanding the user base is critical for evaluating the system’s usability and impact on decision-making processes.

Users engage in several *tasks* with the system. These include *Data collection*, which involves gathering information on the spread of diseases or health conditions; *Analysis*, where users perform statistical and epidemiological analyses; *Data exploration*, includes the use of visual tools to interpret trends and relationships; *Decision-making* leverage system outputs to inform public health policies and responses; and *Collaboration*, that enables users to share data, insights, and reports with other stakeholders. While *Purpose of the System* describes the system’s ultimate function in the public health domain, *Tasks* capture the concrete activities users undertake within the system. Importantly, the same task (e.g., decision-making) may occur across different purposes, though the context and intended outcomes differ.

*Visual Representations* play a central role in these systems, facilitating data interpretation and communication. Common types include *Graphs and charts*(e.g., line, bar, and pie charts) for quantitative comparisons; *Geospatial maps*(e.g., heatmaps and choropleth maps) for spatial pattern recognition; *Network graphs* to illustrate the relationships between variables or events; *time-series plots* for trend analysis over time; and *Dashboards* serving as integrated platforms that showcase multiple visual components simultaneously, enabling users to gain insights at a glance. Lastly, *Hierarchical charts*, such as sunburst charts and treemaps, are effective for exploring data organized in a hierarchical structure.

*Visual analytics techniques* are essential for analyzing and interpreting visual data. Visual analytics is ”the science of analytical reasoning facilitated by interactive visual interfaces” [15]. It supports human decision-making by integrating automated analysis with visualization, enabling the exploration of complex, multivariate data [16]. Various methods are employed within this framework, starting with *statistical and analytical approaches* such as *Descriptive statistics* for summarizing data distribution, *Statistical analysis* involves hypothesis testing for validating patterns, *Regression models* for predictive and explanatory purposes, and *Cluster analysis* is used to detect patterns or hotspots of disease incidence.

*Spatial and geospatial analysis* is another key area that visualizes data across geographic regions to identify hotspots or areas of impact. Techniques such as heatmaps and choropleth maps represent data density or frequency over geographical locations, aiding in the visualization of disease spread. *Geospatial models* incorporate geographical variables to analyze spatial relationships, which are useful for examining disease vectors or environmental factors. *CARTO map* tiles further facilitate interactive exploration of geospatial data.

*Data integration and processing techniques* are essential for system performance. Simplifying the data collection process helps consolidate information from various sources for real-time analysis and interpretation. *Automatic data entry* enhances the efficiency of this process, while frameworks and indices provide standardized indicators or scores for comparing regions or health statuses. *Data normalization and scaling* ensure consistency across diverse datasets, which ultimately improves the comparability of metrics.

The use of *Machine learning and AI* has become invaluable in the realm of visual analytics as well. These systems utilize *Supervised and unsupervised learning* to perform classification, clustering, and anomaly detection in health data. *Deep learning* is employed to process large datasets and complex patterns, such as those used in image recognition in medical diagnostics. *Predictive analytics* helps forecast future disease trends, supports early warning systems, and enhances response planning.

In addition to these techniques, other specialized methods, such as simulation models, optimization algorithms, and interactive dashboards, enhance user engagement and decision-making, ultimately leading to more effective health analytics.

The *interactive analysis capability* refers to the extent to which users can engage with the data. In systems with *high interactivity*, users have full control to manipulate and explore the data through features such as drill-down, zoom, filters, and dynamic updates. *Moderate interactivity* allows for limited exploration, providing some filters and options to adjust views. In contrast, *low interactivity systems* primarily offer static visualizations, giving users minimal control over the data.

Finally, systems are categorized by their implementation status and accessibility. The System/Dashboard category assesses whether the study presents a functional system. A “Yes” indicates a working prototype or operational platform, while a “No” denotes a theoretical or conceptual discussion. The *online availability* category evaluates whether the system or dashboard can be accessed online by external users, which is crucial for reproducibility, transparency, and practical application.

## 3 Results

### 3.1 Study selection

As shown in Fig. 1, the systematic review process began with an initial search across IEEEXplore, ScienceDirect, PubMed, and Google Scholar using relevant keywords. After selecting pertinent papers from these databases, we employed a backward citation chasing approach by examining the reference lists of those papers to identify additional relevant studies. These cited papers (“other sources”) were then subjected to the same screening and selection process.

Initially, our search yielded approximately 13,300 papers from the four databases. Applying a time filter to include only papers published within the last 10 years (2015–2024) reduced the number to 10,552. Limiting the results to English-language papers further reduced the total to 9586.

We then filtered for papers containing visualizations, resulting in 91 papers from IEEEXplore, ScienceDirect, and PubMed. From Google Scholar, we reviewed the first 20 result pages (200 papers), giving a total of 291 papers. We examined the reference lists of these 291 papers using backward citation chasing, identifying 2473 additional papers. After applying the same filtering and selection criteria, this number was narrowed to 359.

Combining the 291 initially selected papers with the 359 identified through reference chasing resulted in 650 papers. After removing duplicates and further screening based on inclusion and exclusion criteria, we finalized a set of 202 papers for the review.

### 3.2 Study characteristics

**Fig. 3:**
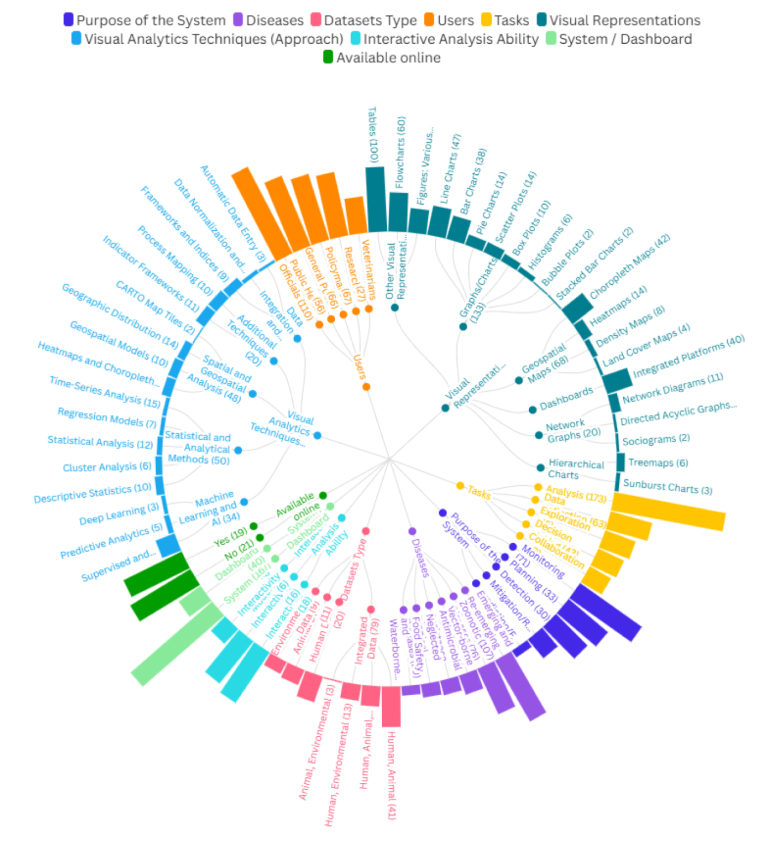
The categories of the classification are sorted by their respective percentages.

In this section, we provide an overview of the characteristics of the studies included in our analysis, as illustrated in Fig. 3. Nine features were assessed, each with sub-categories, and some sub-categories had further divisions. In the following sections, we will investigate the relationships within each feature, between features, and among the subcategories of all the features.

#### 3.2.1 Features characteristics

In terms of the purpose of the system, we found that the most common focus was monitoring, accounting for 56% of the studies, for example, [17], [18], [19], [20], [21]. This was followed by planning, which constituted 38% of the studies [22], [23], [24], [25]. Detection was addressed in 33% of the papers [26], [27], [28], while mitigation and response accounted for 18% [29]. Prediction and forecasting were less emphasized, appearing in only 6% of the studies [23].

The majority of systems focus on Emerging and Re-emerging Diseases, particularly COVID-19, which accounted for 52% of the studies [21], [23], [30], [31], [32]. Other significant areas include zoonotic diseases (37%; [33], [34]), vector-borne diseases (15%; [35]), neglected tropical diseases (10%; [36]), food safety and waterborne diseases (7%; [37]), and antimicrobial resistance (11%; [26]).

Regarding data types, human data were the most frequently used type, appearing in 20% of the studies, e.g., [38], [39], followed by integrated human–animal data in 30% of studies [40], [41], [42]. Environmental data were utilized in 10% of the studies [43], while combinations involving human, animal, and environmental data showed varying levels of integration, accounting for 15% of the studies [44].

The primary users of these systems were public health officials, comprising 53% of the identified user base. This group was prominently represented in several studies, e.g., [39], [45], [46], [47], [48], [49], [50].

Researchers represented 32% of the users, as noted in several studies, e.g., [18], [45], [51], [52], [53], while policymakers accounted for a similar proportion (32%; [23], [25]). The general public comprised 27% of users [35], [52], [54], [55], [56], [57], and veterinarians made up 13% of the user base [58].

Analysis was the dominant task, appearing in 84% of the studies, e.g., [18], [24], [38], [39], [43], [49], [54], [59], [60], [61]. Other commonly reported tasks included data collection (30%; [17], [22], [23]), exploration (22%; [21], [62], [63]), decision-making (21%; [25], [42]), and collaboration (18%; [64], [65]), each playing supportive roles in system functionality.

The most common visual formats were graphs/charts, used in 64% of the studies [18], [24], [60], [62], followed by geospatial maps in 33% of the papers [47], [51]. Dashboards were reported in 20% of the studies [18], [22], [23], while network graphs appeared in 10% [66], and hierarchical charts in 4% [63].

Techniques such as statistical and analytical methods were employed in 35% of the studies, while spatial/geospatial analysis appeared in 32% [18], [22]. Machine Learning/AI approaches were used in 16% of the studies [23], [67]. Other techniques, including data integration and processing, were also presented, but were less dominant.

Most systems provided low to moderate interactivity, with 46% exhibiting low interactivity [25], [66], [68] and 38% moderate interactivity [45], [61], [62]. Fewer systems offered high interactivity, accounting for 16% of the studies [43].

#### 3.2.2 Relationships between features

**Fig. 4:**
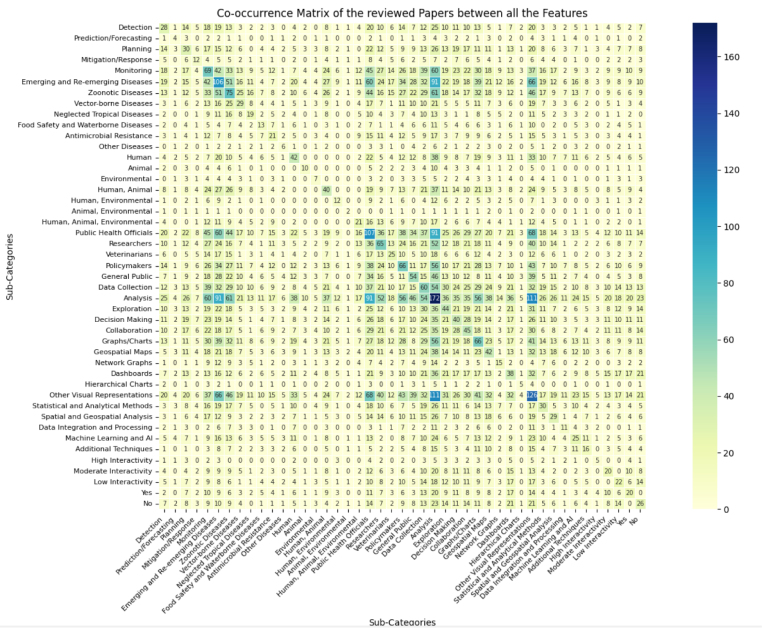
Heatmap illustrating the frequency and strength of relationships among system features. Darker cells indicate stronger associations and higher co-occurrence between features.

Fig. 4 presents the relationships among system features, revealing distinct patterns across purpose, diseases, users, tasks, visualizations, analytical techniques, interactivity, and availability.

Monitoring systems dominate (69 occurrences), primarily addressing emerging and re-emerging diseases (106) and zoonotic diseases (75), while detection systems focus on veterinarians and zoonotic diseases (13). Planning and mitigation/response systems engage policymakers and public health officials, whereas prediction/forecasting systems are rare.

Public health officials are the most frequent users (107), with a focus on analysis (91), while researchers (65) and policymakers (66) are involved in exploration, decision-making, and planning. Veterinarians (25) focus on detection and data collection, and the general public (54) interacts with monitoring systems.

Graphs and charts (64) and geospatial maps (33) are common visualizations, with dashboards appearing in 20% of cases.

Systems generally exhibit low to moderate interactivity, with higher interactivity in research-oriented monitoring tools (25). Statistical and analytical methods (91 occurrences) and spatial/geospatial analysis (32) are prevalent, while ML/AI approaches (24) are applied in exploratory and high-interactivity contexts.

Human and integrated human–animal datasets are most frequent (20%–30%), supporting analysis, exploration, and collaborative tasks. Online availability correlates with higher interactivity and broader user engagement.

Overall, the heatmap highlights a strong focus on monitoring emerging infectious diseases using integrated datasets, task-driven visualizations, and diverse users, emphasizing analysis and decision-making workflows.

### 3.3 Publication trends and venue analysis

**Fig. 5:**
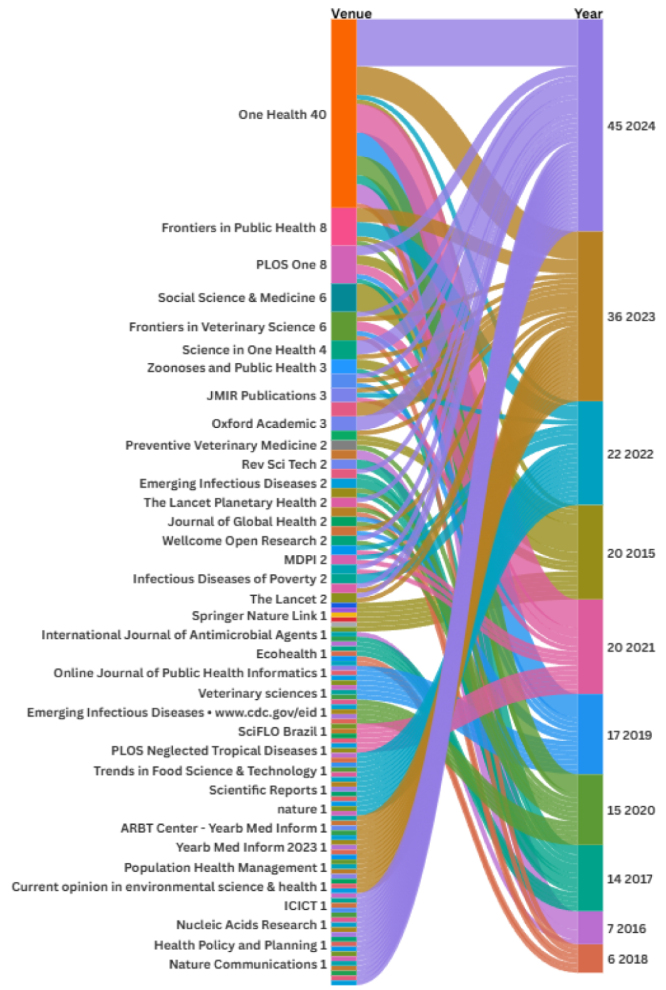
Number of reviewed papers published each year. Venues are listed by publication count, and years are presented in chronological order, from most recent to earliest.

The analysis revealed a significant upward trend in One Health-related publications over the past decade, with a notable surge in 2023 and 2024, as shown in Fig. 5. The journal *One Health* emerges as the dominant publication venue, accounting for 45 papers, underscoring its central role in disseminating research in this field. Other frequently appearing venues include *Frontiers* in Public Health, *PLOS One*, and *Frontiers in Veterinary Science*, reflecting the interdisciplinary nature of One Health scholarship. The increasing diversity of journals and platforms in recent years highlights the expanding relevance and recognition of the One Health approach across public health, veterinary science, environmental health, and informatics. This trend suggests a growing commitment within the scientific community to addressing complex health challenges through integrated, cross-sectoral collaboration.

## 4 Discussion

Understanding the landscape of One Health systems through this analysis provides valuable insights that can inform future development and research by identifying gaps, highlighting effective practices, and setting priorities. Based on the analysis, only a small percentage (6%) of systems focus on prediction/forecasting [23]. This shows a need for future systems to improve predictive capabilities and response strategies, which are crucial for pandemic preparedness and outbreak management. Prior work has emphasized the value of predictive health information systems in epidemic prevention and control. For example, Myers et al. argue that linking environmental data with disease systems could allow for the anticipation of outbreak conditions, thus improving response efforts and resource allocation [69]. Similarly, Keyel et al. highlight the disconnect between academic model development and its application in local public health decision-making, calling for standardized, actionable forecasting tools that can support real-time decisions in vector control and public health planning [70]. Our findings support these observations and suggest that a stronger integration of predictive modeling into One Health systems could address a substantial shortcoming in current implementations.

With only a small proportion of systems using predictive analytics (6%) [59] and machine learning techniques (16%) [71], there is a clear path for growth. Incorporating these advanced methods can improve the ability to forecast disease trends and make data-driven decisions. The presence of spatial and geospatial techniques (32%) [72], [73] underscores the importance of understanding geographic distribution and environmental influences. Future systems can leverage geospatial analysis to identify disease hotspots and track the spread of emerging infectious diseases.

Regarding diseases, the focus is primarily on COVID-19, while other important areas such as zoonotic diseases, vector-borne diseases, and antimicrobial resistance receive less attention. Research should expand to cover these emerging threats to align with the holistic One Health approach. Regarding data integration, few systems use integrated datasets combining human, animal, and environmental data, which is fundamental to One Health. This presents an opportunity to develop systems that better integrate diverse data sources to understand complex disease dynamics.

The popularity of visualizations such as graphs/charts, dashboards, and geospatial maps suggests their effectiveness in communicating data insights. Future systems can continue to use and enhance these visual tools, perhaps combining them for more comprehensive visualization capabilities. Additionally, systems with high or moderate interactivity allow users to engage deeply with the data, emphasizing interactive features (e.g., drill-downs, filtering). In future systems, interactive features can improve user experience and data exploration capabilities, enabling stakeholders to make informed decisions.

The analysis reveals different user types, such as public health officials including healthcare providers, researchers, veterinarians, policymakers, and the general public. This diversity of user groups highlights the need for systems that are tailored to the specific goals and workflows of different stakeholders—offering capabilities such as data collection and export for researchers, decision-support tools for policymakers, and accessible dashboards for public awareness and engagement. Critically, co-development of systems with end users has proven valuable in ensuring systems meet real-world needs, foster adoption, and support sustained use. For instance, the EPIDEMIA system for malaria forecasting was designed through workshops with computer scientists, modelers, and public health partners, leading to successful implementation in Ethiopia’s Amhara region [74]. Similarly, ArboMAP, a system developed for West Nile virus forecasting, emphasized automation and decision-maker usability, allowing public health departments to routinely produce and act on weekly risk predictions [75].

Since most systems emphasize analysis (84%) [72], [76], [77], [78], future tools could also enhance support for tasks like data collection, collaboration, and decision-making. For example, integrating real-time data streams and collaborative features could improve how users work together in pandemic response efforts. With many systems offering only low interactivity, future research can focus on enhancing user interaction with the data. This can be achieved through features such as real-time updates, predictive modeling integration, and dynamic filtering to facilitate deeper analysis. There is an opportunity to adopt or improve techniques like hierarchical charts, network graphs, and flow diagrams, which could offer richer, multidimensional insights into data relationships.

The multidimensional analysis of feature relationships reveals several overarching trends that reflect the current landscape, strengths, and limitations of visualization systems within One Health and pandemic-related research contexts. The patterns identified across second- to higher-order relationships demonstrate not only recurring design choices but also systematic imbalances in how visualization systems are conceptualized, implemented, and targeted toward specific users and diseases.

The findings around disease coverage and user groups can help policymakers identify priority areas for resource allocation. For instance, increased focus on integrating environmental data might better address zoonotic disease outbreaks related to climate change. Enhancing Preparedness and Response Strategies: Insights from monitoring and planning-focused systems can guide the design of tools that not only detect emerging threats but also offer strategic recommendations for outbreak response and long-term planning. We summarized the keys for enhancing One Health systems in Fig. 7.

### 4.1 Limitations

Despite the insights provided by this analysis, several limitations should be acknowledged. First, our review relies on published and publicly available systems, which may introduce selection bias and overlook unpublished or proprietary tools. Furthermore, surveying integrated systems implies that substantial data must be available to form any data-driven conclusions. One Health suggests considering global effects, where a dataset from a specific region or country may not be sufficient to answer the given questions. This inherently excludes factors that might be deemed important for One Health, such as wildlife trade, plants, and food, but indicates that the available datasets are insufficient or unavailable for research to incorporate them into integrated systems. While conceptual frameworks often acknowledge plants as part of ecosystem health [79], few operational systems explicitly integrate plant disease surveillance, agricultural biosecurity, or crop health data [80], [81], [82]. Recent studies have highlighted this omission, calling plant health the “Cinderella of the One Health strategy” and emphasizing its fundamental role in food security, nutrition, and the prevention of downstream impacts on animal and human health [83]. For example, the ongoing threat of *Fusarium Tropical Race 4* (TR4), which endangers global banana production, underscores how neglecting plant health can destabilize food systems and public well-being [84].

Similarly, wildlife trade data, such as those available through the CITES Trade Database, remain underutilized in One Health implementations. These datasets can provide early indicators of zoonotic spillover risks, biodiversity loss, and illegal wildlife exploitation that contribute to emerging disease threats [85]. Integrating plant health surveillance and wildlife trade information into future One Health systems would strengthen the predictive, preventive, and cross-sectoral capacities needed for truly holistic health intelligence.

Lastly, the categorization of system features and tasks, although systematic, may involve subjective interpretation, which could potentially affect reproducibility.

**Fig. 6:**
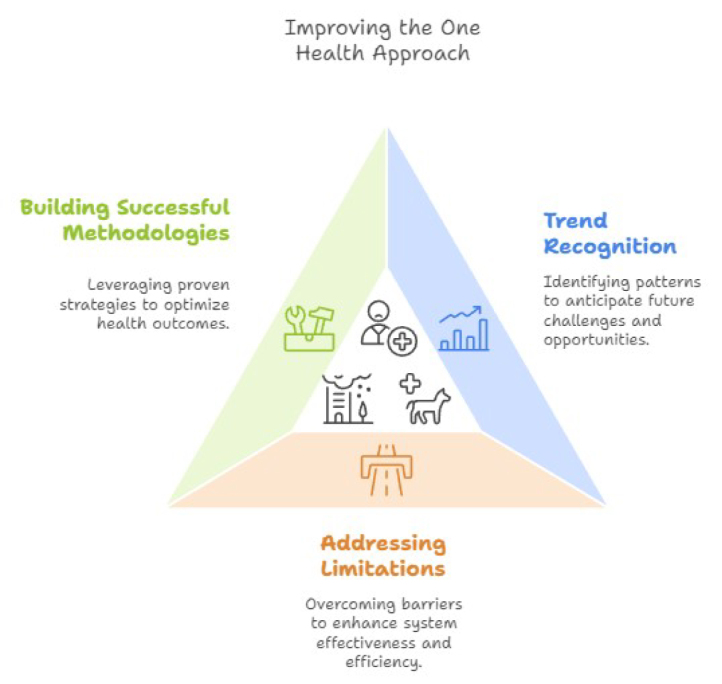
Some suggestions for improving one health approach.

By recognizing trends, addressing limitations, and building upon successful methodologies, future research and development efforts can support the One Health approach more effectively, ultimately improving disease detection, prediction, and prevention strategies across the human–animal–environment interface, as summarized in Fig. 6.

**Fig. 7:**
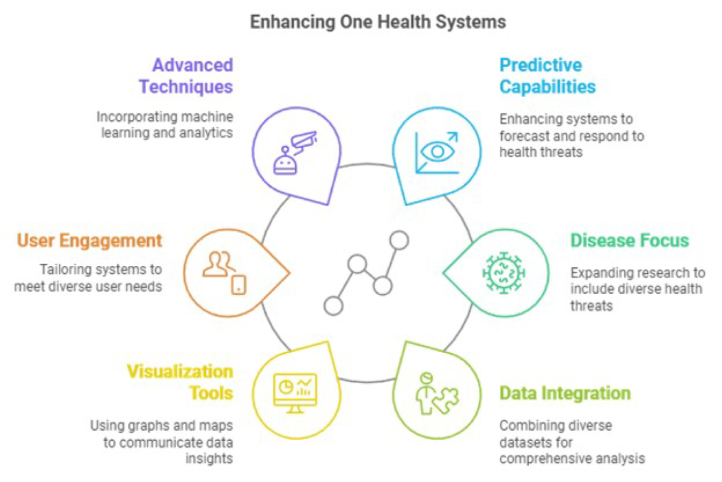
Proposed keys for enhancing one health systems.

## 5 Conclusions

This systematic review examined One Health datasets for the management of infectious diseases. Our paper systematically categorizes and evaluates One Health dashboards and systems based on their use of visualization, visual analytics, types of data, and functionality, aiming to understand their effectiveness in infectious disease management tasks, such as prediction, monitoring, and mitigation. Analyzing systems from 2015 to 2024, the review assessed 202 articles identified through specific keywords in various databases. While a significant portion of studies still rely on single-domain data, there is a clear trend toward integrating multiple datasets, especially Human and Animal data. Fully integrated systems that combine human, animal, and environmental data accounts are limited, and most of these are commentary pieces rather than functional systems. This indicates a growing recognition of the One Health framework, but also highlights a gap—and an opportunity—for more robust, applied systems that truly integrate data across all three domains. Based on the comprehensive review, we believe that our paper is unique in its focus and scope compared to existing literature on similar topics.

## CRediT authorship contribution statement

**Aseel Basheer:** Writing – review & editing, Writing – original draft, Visualization, Validation, Software, Resources, Project administration, Methodology, Formal analysis, Data curation. **Matthew Tran:** Writing – original draft, Software, Methodology. **Baseer Khan:** Writing – original draft, Software, Methodology. **Wolfgang Jentner:** Writing – review & editing, Writing – original draft, Validation, Supervision, Methodology, Visualization. **Aaron Wendelboe:** Writing – review & editing, Writing – original draft, Validation, Methodology. **Jason Vogel:** Writing – review & editing, Writing – original draft, Validation, Methodology. **Katrin Kuhn:** Writing – review & editing, Writing – original draft, Validation, Methodology. **Michael C. Wimberly:** Writing – review & editing, Writing – original draft, Validation, Methodology. **David Ebert:** Writing – review & editing, Writing – original draft, Validation, Supervision, Methodology, Visualization.

## Declaration of competing interest

Aseel Basheer reports financial support was provided by National Science Foundation. If there are other authors, they declare that they have no known competing financial interests or personal relationships that could have appeared to influence the work reported in this paper.

Wolfgang Jentner reports financial support was provided by National Science Foundation. If there are other authors, they declare that they have no known competing financial interests or personal relationships that could have appeared to influence the work reported in this paper.

Aaron Wendelboe reports financial support was provided by University of Arkansas for Medical Sciences. If there are other authors, they declare that they have no known competing financial interests or personal relationships that could have appeared to influence the work reported in this paper.

Jason Vogel reports financial support was provided by University of Oklahoma. Jason Vogel reports financial support was provided by University of Oklahoma. Jason Vogel reports a relationship with University of Oklahoma that includes:. If there are other authors, they declare that they have no known competing financial interests or personal relationships that could have appeared to influence the work reported in this paper.

Michael C. Wimberly reports financial support was provided by The University of Oklahoma. Michael C. Wimberly reports a relationship with The University of Oklahoma that includes: funding grants. If there are other authors, they declare that they have no known competing financial interests or personal relationships that could have appeared to influence the work reported in this paper.

Matthew Tran, Baseer Khan, Katrin Kuhn and David Ebert declare that they have no known competing financial interests or personal relationships that could have appeared to influence the work reported in this paper.

## Data Availability

OSF Registry: https://osf.io/zvue2/.
